# The deubiquitinase UCHL1 negatively controls osteoclastogenesis by regulating TAZ/NFATC1 signalling

**DOI:** 10.7150/ijbs.82152

**Published:** 2023-04-24

**Authors:** Zhenhua Feng, Siyue Tao, Zhaobo Huang, Bingjie Zheng, Xiangxi Kong, Yufeng Xiang, Qibin Zhang, Haixin Song, Zhikun Xu, Xiaoan Wei, Fengdong Zhao, Jian Chen

**Affiliations:** 1 Department of Orthopaedic Surgery, Sir Run Run Shaw Hospital, Zhejiang University School of Medicine, Hangzhou, China; 2 Key Laboratory of Musculoskeletal System Degeneration and Regeneration Translational Research of Zhejiang Province, Hangzhou, China

**Keywords:** UCHL1, TAZ, Osteoclast, NFATC1, osteoporosis

## Abstract

The ubiquitin‒proteasome system (UPS) plays a key role in maintaining protein homeostasis and bone remodelling. However, the role of deubiquitinating enzymes (DUBs) in bone resorption is still not well defined. Here, we identified the deubiquitinase ubiquitin C-terminal hydrolase 1 (UCHL1) as a negative regulator of osteoclastogenesis by using the GEO database, proteomic analysis, and RNAi. Osteoclast-specific UCHL1 conditional knockout mice exhibited a severe osteoporosis phenotype in an ovariectomized model. Mechanistically, UCHL1 deubiquitinated and stabilized the transcriptional coactivator with PDZ-binding motif (TAZ) at the K46 residue, thereby inhibiting osteoclastogenesis. The TAZ protein underwent K48-linked polyubiquitination, which was degraded by UCHL1. As a substrate of UCHL1, TAZ regulates NFATC1 through a nontranscriptional coactivator function by competing with calcineurin A (CNA) for binding to NFATC1, which inhibits NFATC1 dephosphorylation and nuclear transport to impede osteoclastogenesis. Moreover, overexpression of UCHL1 locally alleviated acute and chronic bone loss. These findings suggest that activating UCHL1 may serve as a novel therapeutic approach targeting bone loss in various bone pathological states.

## Introduction

There are two stages of bone remodelling: bone resorption and bone formation. Osteoclasts remove old or damaged bone, and osteoblasts rebuild new bone. Excessive bone resorption contributes to various bone diseases, such as rheumatoid arthritis, bone erosion, and osteoporosis 1-3. Osteoclasts are multinuclear giant cells that degrade the bone matrix, resulting in impaired bone microarchitecture and mechanical properties 4, 5. The mechanism by which osteoclast differentiation is regulated must be further explored to develop new therapeutic targets for osteoporosis.

The ubiquitin‒proteasome system (UPS) is mainly composed of deubiquitinase enzymes (DUBs), Ub-activating enzyme (E1), Ub-conjugating enzymes (E2s), Ub-protein ligating enzymes (E3s), 26S proteasome, and Ub. The UPS plays an important role in maintaining protein homeostasis and bone remodelling 6, 7. Nevertheless, the role of DUBs in bone resorption is still not well defined. Therefore, screening the critical DUBs during osteoclastogenesis by high-throughput methods and elucidating the underlying mechanism are necessary. Ubiquitin C-terminal hydrolase 1 (UCHL1), enriched in neuronal tissues, is a deubiquitinating enzyme (DUB) responsible for protein turnover 8, 9. Recently, UCHL1 was identified to function in cardiovascular diseases and other nonneurological diseases 10-12. UCHL1 targets epidermal growth factor receptor (EGFR) ubiquitination and promotes its stability to regulate cardiac hypertrophy 11. UCHL1 can also remove the ubiquitination of CD36 to promote foam cell formation 13. UCHL1 has been identified as an oncogenic protein that promotes TGFβ induction of breast cancer metastasis 14. Previous studies reported that UCHL1 affects skeletal muscle growth and function 15, 16. Shim et al. used gracile axonal dystrophy (gad) mice that mimic global UCHL1 knockdown, leading to reduced bone mineralization and bone mass 17. Coudert et al. reported that UCHL1 is upregulated in autosomal dominant osteopetrosis type II patients 18. However, whether and how UCHL1 functions in osteoclasts and leads to skeletal abnormalities are poorly understood.

WW domain-containing transcription regulator protein 1 (WWTR1, also known as TAZ) is the major effector of the Hippo pathway. Previous studies have shown that TAZ, as a transcription coregulator, interacts with various transcription factors, such as RUNX2, TEAD, and PPARγ 19-21. Increasing evidence has demonstrated that TAZ is involved in bone remodelling balance 22. TAZ, as a transcriptional coregulator, promotes osteogenic differentiation by promoting runx2 transcription and inhibits adipose differentiation by inhibiting PPARγ-dependent transcription 23. Recent studies have suggested that TAZ plays a new regulatory role in osteoclast formation 24. Furthermore, protein activation, trafficking, and stability are controlled by posttranslational modifications such as acetylation, SUMOylation, phosphorylation, and ubiquitination 25, 26. Nevertheless, the regulatory mechanism of posttranslational modification of TAZ in osteoclasts remains to be explained.

In the present study, we found that UCHL1 acted as a negative regulator of osteoclastogenesis by removing TAZ ubiquitination and regulating TAZ/NFATC1 signalling. Osteoclast-specific UCHL1 knockout resulted in significantly reduced bone mass *in vivo* induced by ovariectomy (OVX) compared to wild-type litters. UCHL1 overexpression locally relieves acute and chronic bone loss. Taken together, our findings suggest that UCHL1 activation may be a novel treatment strategy for a variety of acute and chronic bone loss conditions.

## Materials and Methods

### Reagents

Alpha-MEM, DMEM, FBS and Opti-MEM were purchased from Gibco. RANKL was purchased from R&D. Recombinant mouse M-CSF was purchased from Novoprotein. Chloroquine diphosphate (CQ) was purchased from APExBIO, MG-132 was purchased from MCE, Thapsigargin was purchased from APExBIO MCE.

### Mice

All animal experiments were approved by the Ethics Committee of Sir Run Run Shaw Hospital, Zhejiang University School of Medicine. UCHL1 flox/flox mice and LysM-Cre mice were obtained from Gempharmatech Co., Ltd. In all our experiments, The same sex litters of UCHL1 /flox were used as control.

### Cell culture

The humidified environment of 5% CO2 and 95% air at 37°C were used in cell culture. BMMs were isolated by marrow flushing of femora and tibia of mice as our previous protocol 27. Isolated BMMs were cultured in alpha-MEN (10% FBS and 100 U/ml penicillin-streptomycin) supplemented with 25 ng/ml M-CSF. To generate osteoclasts, BMMs gave additional 50 ng/ml RANKL stimulation. At the end of osteoclast culture, we fixed the cells in 4% paraformaldehyde for 15 min and stained them in TRAP solution for 1 h.

### Bone resorption experiment

The bone resorption experiment was conducted as previously described 28. 96-well hydroxyapatite-coated plates were used to inoculate BMM cells (#3989, Corning Inc., USA). Osteoclasts were cultured in medium containing MCSF (25 ng/ml) and RANKL (50 ng/ml) for 3 days to generate osteoclasts. 10% sodium hypochlorite was used to clean out the cells from the well plate. The area of bone resorption pits was photographed under an optical microscope and measured by Image J software.

### Micro-CT scanning

The tibia and lumbar vertebrae of mice were scanned using the Skyscan 1072 micro-CT system after paraformaldehyde fixation. Tibias and calvarias in figure 6 were scanned using the Skyscan 1275 micro-CT system. We used a 0.5 mm bone trabecula from the growth plate for qualitative and quantitative analysis. BV/TV, Tb.Sp, Tb.Th, and Tb.N was determined for each sample, as reported previously 27.

### siRNA transfection

The transfection system was as previously reported 29. siRNAs for UCHL1 and TAZ were obtained from RiboBio. Briefly, BMMs were inoculated in well plates for 24 hours and siRNAs were transfected using Lipofectamine™iMAX transfection reagent. The siRNA sequences for mouse UCHL1-siRNA-1 (Sequence: 5'-GCAGCUAUUAGGGAACAAGUU-3'), UCHL1-siRNA-2 (Sequence: 5'-CCAGUGAUUGUGGAGACAUUU-3'), human UCHL1-siRNA (Sequence: 5'-GGACAAGAAGTTAGTCCTA-3'), mouse TAZ-siRNA (Sequence: 5'-GATGAATCCGTCCTCGGTG-3').

### Western blot analysis

The cells were mixed with a RIPA buffer containing 100 mM phenyl methane sulfonyl fluoride (PMSF) and a phosphatase inhibitor and incubated on ice cubes, then centrifuged at 12,000 rpm for 15 min to separate the supernatant for the next step. SDS-PAGE electrophoresis were performed according to the formerly protocol 30. Protein bands were prepared by goat-anti-rabbit or goat-anti-mouse immunoglobulin G (Abcam) and then detected with ECL reagent in Amersham Imager 600 (GE, USA). UCHL1 rabbit mAb (#13179, CST, USA, 1:1000), GAPDH Mouse mAb (#AC002, ABclonal, USA, 1:1000), Flag-tag rabbit mAb (#14793S, CST, USA, 1:1000), TAZ Mouse mAb (#ab242313, Abcam, UK, 1:1000), Myc-tag rabbit pAb (#R1208-1, HUABIO, China, 1:1000), Phospho-serine/threonine(p-S/T) (PM3801, ECM Bioscience, USA, 1:2000), NFATC1 Mouse mAb (#sc-7294, Santa Cruz, USA, 1:1000). All full-length western blots were illustrated in Supplementary Material.

### RNA extraction and quantitative real-time PCR

We isolated mRNA from cells using an Ultrapure RNA Kit (#CW0581, CWBIO, China). HiFiScript cDNA Synthesis kit (#CW2569, CWBIO, China) and UltraSYBR Mixture (#CW0957, CWBIO, China) were used for mRNA reverse transcription and RT-qPCR. Primer sequences are presented in Supplementary Table 1.

### Co-immunoprecipitation (IP) assay

Both primary cells and 293T cells were used for co-IP assay. Cells were lysed with a lysate buffer containing a cocktail of 1mm PMSF, 1mm dithiothreitol (DTT), and a protease inhibitor. Cell lysates were immunoprecipitated with anti-UCHL1 (#ET1702-83 HUABIO, China, 1:100), anti-Flag (#14793S, CST, USA, 1:100), anti-TAZ (#83669, CST, USA, 1:100) at 4°C overnight, then incubate it with protein A/G-beads for 4 h at 4 °C. Subsequently, the complex was cleaned 5 times with PBS containing the protease inhibitor at 4°C. Binding proteins were isolated with 10% SDS buffer and analyzed by WB.

### Immunofluorescence staining assay

The treated cells were fixed with 4% paraformaldehyde for 10 min, permeated with 0.1% Triton X-100 for 5 min, sealed with 5% goat serum for 1 hour, and incubated with UCHL1 and TAZ primary antibodies overnight at 4 ℃. Incubate with goat anti-rabbit secondary antibody or goat anti-mouse secondary antibody for 30 min, respectively, and nuclear stained with 0.1 μg/mL DAPI for 10 min. The images were taken by microscope (Nikon, Japan).

### Ovariectomy (OVX)-induced osteoporosis model

A chronic bone loss model induced by ovx was established as described previously 31. Prosthetic oophorectomy or bilateral oophorectomy were performed on 12-week-old female cKO mice or their litters. For UCHL1-AAV rescue experiments, OVX or sham mice were injected via the intramedullary route with AAV expressing GFP or UCHL1 (Hanbio, China) in both femurs and tibias. The mice recovered from ovariectomy for six weeks and then sacrificed for the experiment. The femur and tibia were collected and fixed with 4% paraformaldehyde for microscopic ct and bone morphometric analysis.

### LPS-induced calvarial osteolysis model

LPS-induced acute bone-loss model was established as described previously 32. For UCHL1-AAV rescue experiments, 12-week-old male C57BL/6 mice were divided into three groups: Sham, LPS+AAV-Vector, and LPS+AAV-UCHL1. Mice were anesthetized with pentobarbital and injected subcutaneously with 25 mg/kg LPS. 7 days after modeling, skull was collected and fixed with 4% paraformaldehyde for micro-CT and bone morphology determination.

### Statistical Analysis

The datasets are represented as mean ± SD. Statistical analyses were performed using SPSS 19.0 (SPSS, Chicago). Statistical differences were analyzed by two-tailed Student's t-test or one-way ANOVA followed by Tukey's post hoc analyses where appropriate. For those non-normally distributed data, nonparameter tests were used. P value ≤ 0.05 was considered statistically significant.

## Results

### UCHL1 silencing promotes osteoclast formation

**Figure 1:**
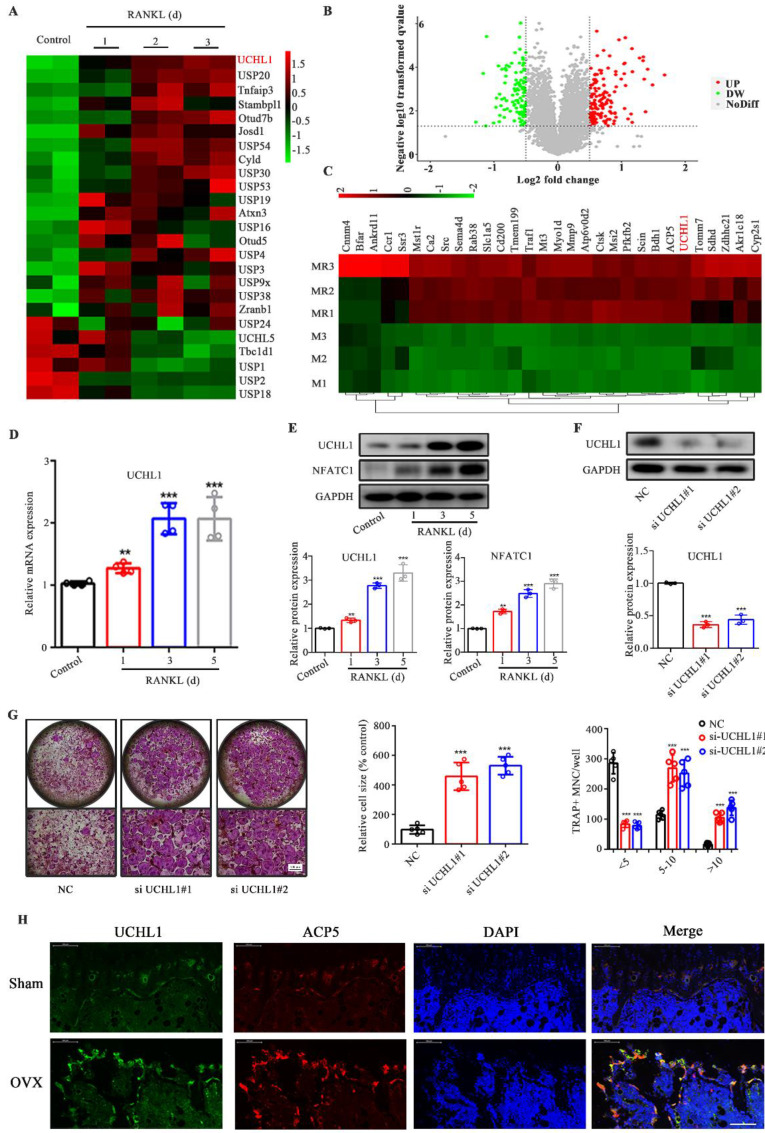
UCHL1 is up-regulated in osteoclastogenesis and inhibits osteoclast formation. A) The cluster of deubiquitinase gene expression profiles in RANKL-induced mouse BMMs at days 0, 1, 2, and 3. B) Volcanograms of differentially expressed proteins stimulated and unstimulated by RANKL in mouse BMMs. C) Top 30 proteins upregulated in RANKL stimulated mouse BMMs. D) The mRNA levels of UCHL1 in different stages of osteoclast formation compared with day 0 analyzed by RT-qPCR assay. E) The protein level of UCHL1 and NFATC1 during osteoclast formation. F) UCHL1 expression in negative control-infected BMMs (NC) and BMMs infected with siRNA targeting UCHL1(si UCHL1). G) TRAP staining to detect osteoclastogenesis of NC, si-UCHL1#1 and si-UCHL1#2 BMMs. Scale bars, 500 μm. The size and number of nuclei of trap - positive multinucleated cells. (N=5). H) Immunofluorescence of UCHL1(green) and ACP5(red) from femur sections of control and OVX mice. Scale bars, 100 μm. Data are presented as mean ± SD. *P < 0.05, **P < 0.01, ***P < 0.001.

Given the limited attention attributed to the investigation of ubiquitination in osteoclastogenesis, we analysed the transcriptome data available through the Gene Expression Omnibus (GEO) database (GSE57468), focusing on DUBs. Among the differential DUBs in osteoclasts, 19 were upregulated and 6 were downregulated, of which UCHL1 was the most obviously upregulated (Figure 1A). To investigate the expression of the DUB family in osteoclasts, we collected RANKL-stimulated mouse bone marrow macrophages (BMMs) for 0 and 3 days for quantitative proteomic analysis. Figure 1B shows volcano plots of unaffected and differentially expressed proteins in RANKL-treated macrophages. The top 30 upregulated proteins are shown in Figure 1C, except for osteoclast-associated proteins (ACP5, MMP9, Atp6v0d2, CTSK, etc.), UCHL1 is the only DUB. qPCR results confirmed increased UCHL1 mRNA expression in RANKL-treated bmm cells in a time-dependent manner (Figure 1D). Similar to NFATC1, UCHL1 protein expression was upregulated in a time-dependent manner with RANKL treatment (Figure 1E). These data suggest that UCHL1 is involved in the process of osteoclast differentiation.

Hence, we silenced UCHL1 expression in BMMs by transfection with siRNA. Compared with NC, western blotting confirmed the knockout efficiency (Figure 1F). Interestingly, our data suggest that UCHL1 silencing strongly increases osteoclast formation (Figure 1G). Immunofluorescence results showed that the levels of UCHL1 and tartrate-resistant acid phosphatase type 5 (ACP5) in OVX mouse femurs was significantly higher than that in the normal control group, and there was colocalization. (Figure 1H). These data indicated that UCHL1 might be a negative regulator of osteoclastogenesis.

### Loss of UCHL1 exacerbates OVX-induced bone loss in mice and hyperactivates osteoclasts *in vitro*

To determine whether UCHL1 is involved in osteoclast formation and activity, we generated LysM-Cre-UCHL1 conditional knockout (cKO) mice through specific deletion of UCHL1 in osteoclast precursors. The deletion of UCHL1 in LysM-Cre-UCHL1 BMMs was confirmed by quantitative PCR and immunoblotting (Supplementary Figure 1B, C). Mouse genotypes were identified by PCR (Supplementary Figure 1A).

**Figure 2:**
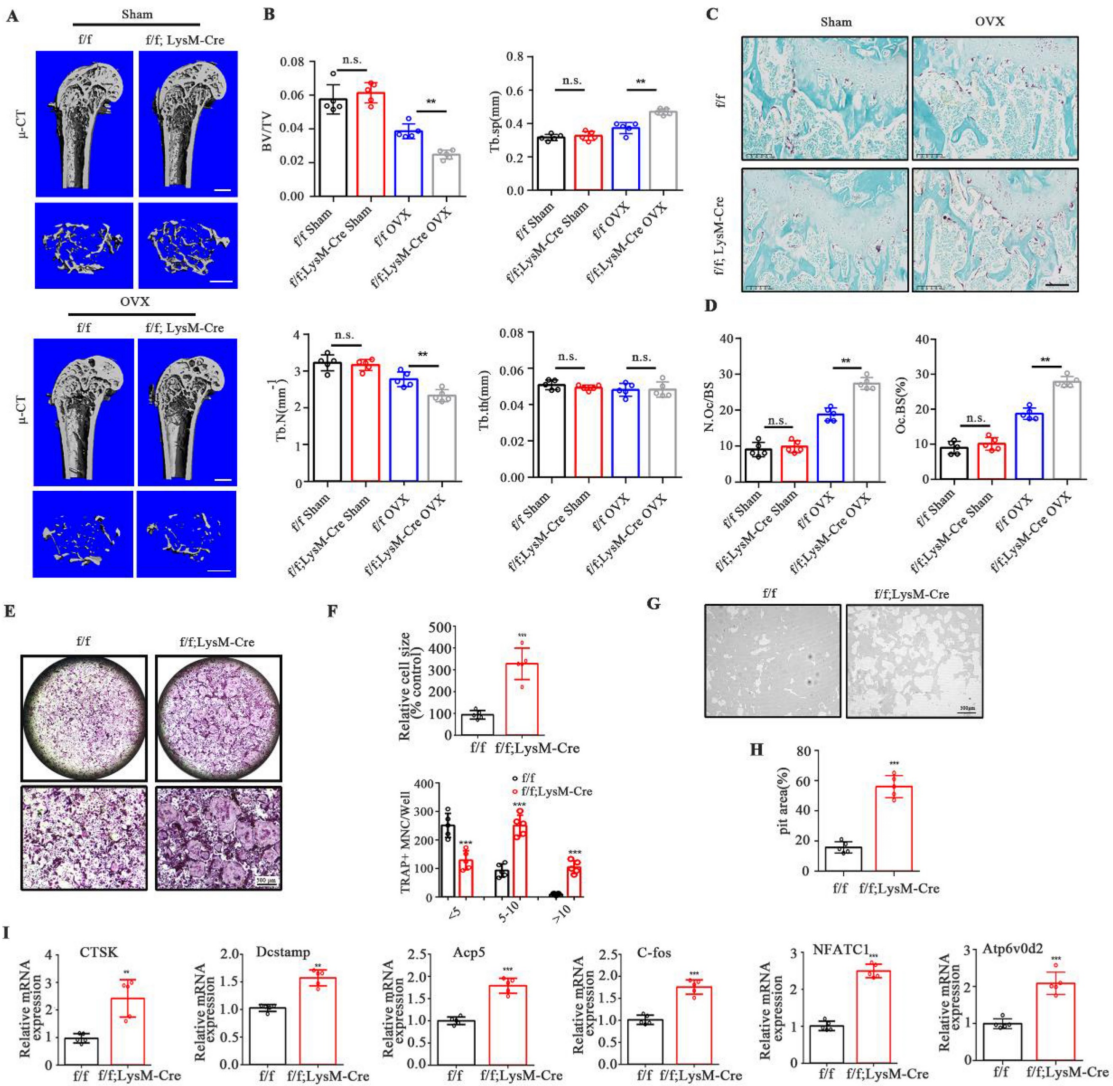
Osteoclast-specific UCHL1-CKO mice display severe osteoporosis in the OVX model and promotes osteoclastogenesis *in vitro*. A) Micro-CT image and three-dimensional (3D) reconstruction images of the distal femur from female UCHL1 CKO or wild-type littermates mice that received sham or ovariectomy surgery for 6 weeks. Scale bars, 1 mm. B) Quantification of bone volume/ tissue volume (BV/TV), trabecular separation (Tb.Sp), trabecular number (Tb.N), and trabecular thickness (Tb.Th) (n=5). C) TRAP staining of sections from the four groups. Scale bars, 200 μm. D) Quantification of osteoclast number/bone surface (N. Oc/BS) and the percentage of osteoclast surface per bone surface (Oc. S/BS (%)) (n=5). E) TRAP staining to detect osteoclastogenesis of BMMs from UCHL1 cKO and wild-type littermates mice. Scale bars, 500 μm. F) Quantification of size and nuclei numbers of TRAP-positive multinuclear cells (n = 5). G, H) Microscope images and quantification of the relative pit resorption size of hydroxyapatite-coated plates (n=5). Scale bars, 500 μm. I) RT-qPCR analysis of CTSK, Dcstamp, Acp5, C-fos, NFATC1, and Atp6vod2 in UCHL1 cKO and wild-type littermates osteoclasts. (n=5). Data are presented as mean ± SD. *P < 0.05, **P < 0.01, ***P < 0.001.

No significant morphological changes were observed in LysM-Cre-UCHL1 and WT litters at 12 weeks of age. μ-CT showed no increase or decrease in bone mass and cortical bone of the femur and lumbar trabecula in the two groups (Supplementary Figure 2A-F). To further unveil the role of UCHL1 in bone homeostasis, OVX surgery was performed to investigate events under pathological conditions. Quantitative bone morphological parameter evaluation revealed a severe osteoporosis phenotype in UCHL1 cKO-OVX mice, with low bone volume/tissue volume (BV/TV) and trabecular number (Tb. N) and elevated trabecular separation (Tb. Sp) (Figure 2A and B). Bone mass analysis of the second lumbar vertebra also demonstrated severe bone loss in UCHL1 cKO mice after OVX surgery (Supplementary Figure 3A and B). Compared with WT litters, the femoral tartrate-resistant acid phosphatase (TRAP) activity of UCHL1 cKO mice was significantly increased after OVX (Figure 2C, D).

We investigated the effects of RANKL on osteoclastogenesis in the absence of UCHL1 and found that UCHL1 cKO significantly promoted osteoclast formation (Figure 2E), with an increased number and size of TRAP-positive cells (Figure 2F). To further analyse the role of UCHL1 in osteoclastic bone resorption, we conducted bone resorption experiments *in vitro* and found that the bone resorption pits of UCHL1 cKO osteoclasts were larger than those of WT cells (Figure 2G and H). Further RT‒qPCR analysis revealed that osteoclast-encoding genes, such as cathepsin K (Ctsk), Dc-stamp, Acp5, C-fos, nuclear factor of activated T cells 1 (NFATC1) and Atp6v0d2, were higher in UCHL1 cKO than in WT BMMs cultured with M-CSF and RANKL for 3 days (Figure 2I). These data demonstrated that UCHL1 deficiency exacerbated bone loss under pathological conditions by promoting osteoclast formation.

### UCHL1 protects TAZ against degradation to inhibit osteoclastogenesis

**Figure 3:**
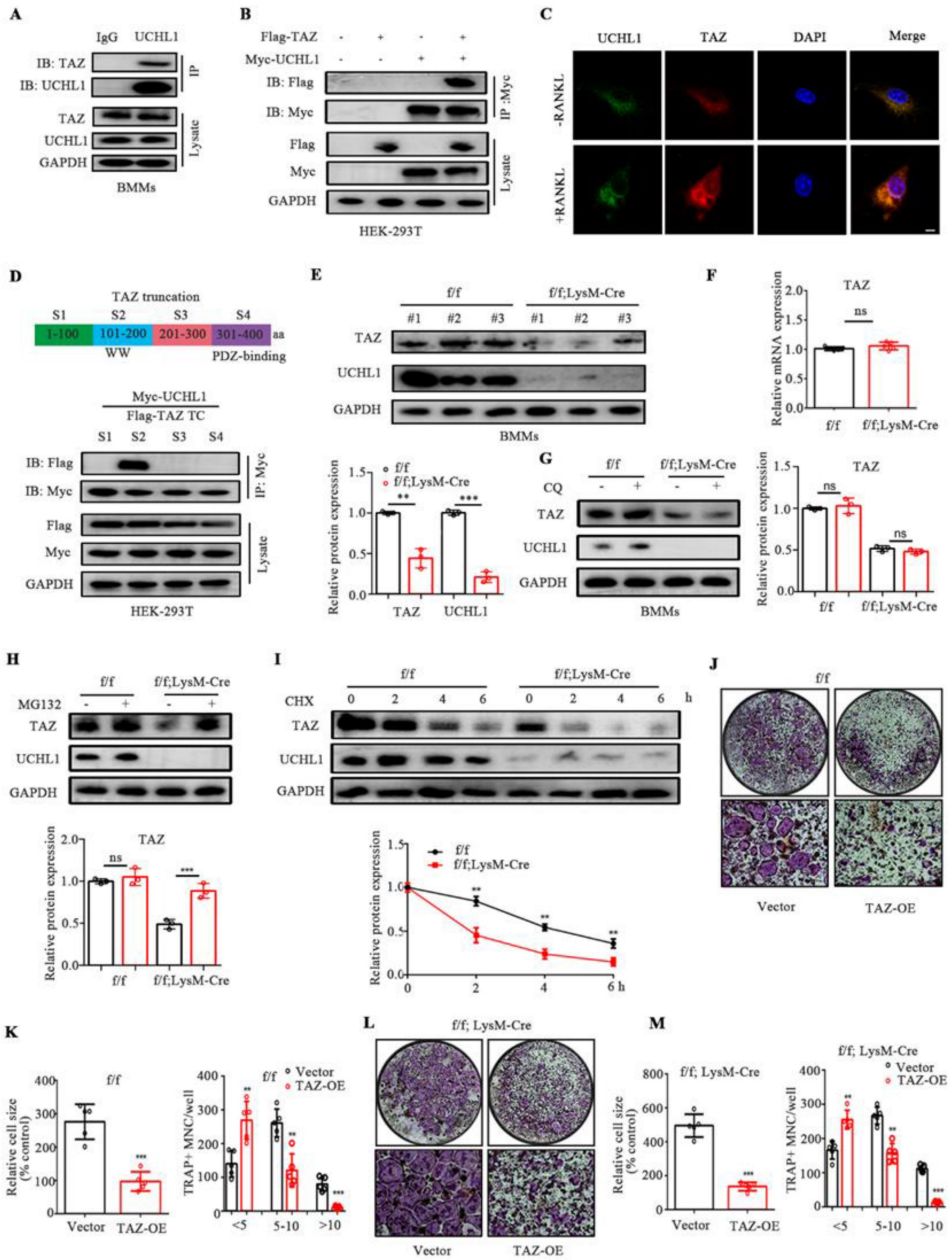
TAZ acts as a substrate of UCHL1 and rescue the effect of UCHL1 knockout on osteoclasts. A) Endogenous protein interaction between UCHL1 and TAZ in BMMs was examined by immunoprecipitate with anti-rabbit IgG or anti-UCHL1 antibody, and analyzed by WB with antibodies to detect UCHL1 and TAZ. B) Exogenous protein interaction between UCHL1 and TAZ was demonstrated in HEK-293T. C) Colocalization between UCHL1 (Green) and TAZ (Red) in unstimulated or RANKL stimulated BMMs by confocal microscopy. Scale bars, 10 μm. D) Full-length and various truncated fragments of TAZ. S1-S4 and UCHL1 plasmids were co-transfected in HEK-293T cells. The interactions were detected through CO-IP analyses. E) The protein level of TAZ in UCHL1 cKO and wild-type littermates BMMs. F) The mRNA levels of TAZ in UCHL1 cKO and wild-type littermates BMMs. G, H) Western blots showing TAZ expression in UCHL1 cKO and wild-type littermates BMMs treated with chloroquine (CQ) or MG132. I) The protein levels of UCHL1 and TAZ in UCHL1 cKO and wild-type littermates BMMs treated with CHX (10 μm) for the indicated time periods. J, L) TRAP staining to detect osteoclastogenesis of BMMs from UCHL1 flox/flox or UCHL1 cKO mice transfected with TAZ overexpressed adenovirus or vector. Scale bars, 500 μm. K, M) Quantification of size and nuclei numbers of TRAP-positive multinuclear cells (n = 5). Data are presented as mean ± SD. *P < 0.05, **P < 0.01, ***P < 0.001.

Mussell et al. used the Dharmacon siRNA DUB library to screen deubiquitinating enzymes that alter the TAZ protein and found that TAZ may be the substrate of UCHL1, but the specific binding effect and mechanism of the two proteins have not been further confirmed and elucidated 33. To investigate whether UCHL1 is associated with TAZ, we conducted a co-IP experiment and found that antibodies against UCHL1 effectively precipitated TAZ in BMMs, while control IgG did not (Figure 3A). Human embryonic kidney (HEK)-293T cells were used for the Co-IP assay. Exogenous MYC-tagged UCHL1 also coprecipitated with FLAG-tagged TAZ. (Figure 3 B). Next, we carried out immunofluorescence staining of UCHL1 and TAZ in BMM cells stimulated with or without RANKL. Immunofluorescence colocalization showed that both UCHL1 and TAZ were mainly distributed in the cytoplasm, indirectly suggesting an interaction between UCHL1 and TAZ (Figure 3C). We then tried to identify specific regions in UCHL1 and TAZ that are critical to their interaction. We generated four TAZ truncations according to its functional domain (Homo sapiens). By co-IP assays, we found that Segment 2 of TAZ (amino acids 101-200) coimmunoprecipitated with UCHL1 (Homo sapiens, Figure 3D).

We noticed that UCHL1 depletion in BMMs profoundly decreased TAZ protein levels without affecting TAZ mRNA expression (Figure 3E and F). MG132, a proteasome inhibitor, restored the reduced TAZ levels upon UCHL1 knockdown, while the lysosome inhibitor chloroquine (CQ) failed to restore TAZ protein expression (Figure 3G and H). Compared with the control group, UCHL1 knockout significantly reduced the half-life of the TAZ protein, suggesting that UCHL1 regulates TAZ homeostasis by blocking TAZ proteasome-mediated degradation (Figure 3I).

Since our previous data showed that UCHL1 interacts with TAZ and stabilizes it, we next investigated whether UCHL1 inhibits osteoclast formation by selectively targeting TAZ. We infected BMM cells with adenovirus TAZ or empty vector GFP (Ad-GFP) with or without UCHL1 deletion. We overexpressed TAZ in WT and UCHL1 cKO BMMs using adenovirus. In the control group, the presence of infection was assessed by green fluorescent protein expression (Supplementary Figure 4A), and TAZ expression was confirmed by WB (Supplementary Figure 4B). TRAP staining showed that overexpression of TAZ not only inhibited the osteoclast differentiation of normal BMM cells but also significantly inhibited the enhanced osteoclast differentiation caused by UCHL1 knockout (Figure 3 J-M). These results demonstrate that UCHL1 regulates RANKL-induced osteoclastogenesis by increasing TAZ stability.

### UCHL1 mediates the removal of K48-linked poly-Ub chains on TAZ

**Figure 4:**
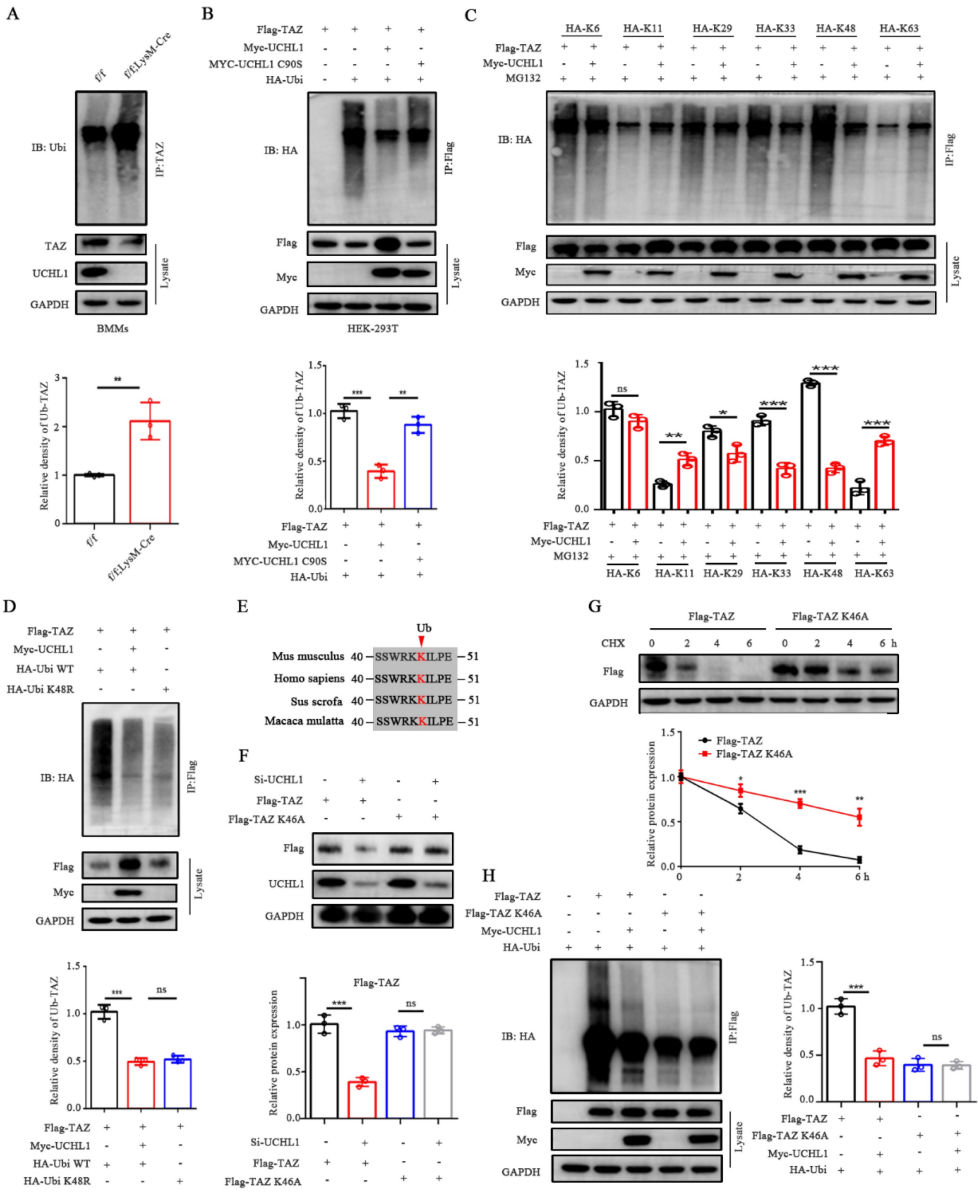
UCHL1 mediates the deubiquitination of TAZ. A) IB analysis of the ubiquitination of TAZ in UCHL1 cKO BMMs. Bmms lysates of both groups were IP treated with anti-TAZ, and their ubiquitization (Ub) levels were analyzed with IB. B) HEK-293T cells were transfected with HA-tagged ubiquitin along with the Flag-TAZ and Myc-tagged UCHL1 or Myc-tagged UCHL1 C90S overexpressed plasmids. HEK-293T lysates were IP with anti-Flag antibody and ubiquitin of TAZ was detected with anti-HA antibody. C) HEK-293T cell lysates transfected with various HA-tagged ubiquitin mutants (including K6, K11, K29, K33, K48 and K63) and corresponding overexpressed plasmids were treated with MG132 before harvest to obtain anti-Flag antibody IP, and then ubiquitin was detected by IB. D) HEK-293T cell lysates and corresponding overexpressed plasmids were transfected with HA-tagged Ubi WT or HA-tagged Ubi K48R, IP with anti-FLAG antibody, and then the ubiquitin of TAZ was detected with anti-HA antibody. E) Sequence alignment of Ub loci in TAZ histogram of different species. F) Cells were transfected with truncated TAZ lysine mutant (TAZ-K46A) and UCHL1 for WB. G) The expression of Flag-TAZ HEK-293T cells transfected with Flag-tagged TAZ or Flag-tagged TAZ K46A mutant plasmids in treated with CHX (10 μm) for the indicated time periods. H) IB analyses of deubiquitination of TAZ or TAZ K46A mutant in UCHL1 overexpressed HEK-293T cells. Data are presented as mean ± SD. *P < 0.05, **P < 0.01, ***P < 0.001.

Since UCHL1 belongs to the DUB family, we investigated whether it regulates TAZ ubiquitination in BMMs. As expected, UCHL1 cKO BMMs showed significantly increased TAZ ubiquitination compared to UCHL1f/f controls (Figure 4A). To characterize the effect of UCHL1 on TAZ ubiquitination, HEK-293T cells were cotransfected with Flag-tagged TAZ, Myc-tagged UCHL1, either WT or mutants without deubiquitinase activity (C90S), and HA-tagged ubiquitin. The data showed that UCHL1-WT deubiquitinated TAZ, but UCHL1-C90S did not (Figure 4B). We further investigated which ubiquitin chain isoforms in HEK-293T cells are coupled to TAZ and regulated by UCHL1. As illustrated in Figure 4C, UCHL1 selectively removed the K29-, K33- and K48-linked ubiquitin chains from TAZ and conversely increased the K11- and K63-linked ubiquitin chains. UCHL1 had the most obvious downregulation effect on the K48-linked ubiquitin chains. To further verify the specificity of ubiquitin chain types, we transfected HEK-293T cells with HA-tagged wild-type (WT) or K48R monosense mutant ubiquitin chain overexpression plasmids. Co-IP experiments showed that TAZ could be modified by the wild-type ubiquitin chain and that its ubiquitin level could be downregulated by UCHL1. The ubiquitination level of TAZ was downregulated after transfection of the K48R mutant compared with wild-type ubiquitin chains (Figure 4D). These data support our conclusion that K48 polyubiquitin chains are involved in the ubiquitination of TAZ, and this specific ubiquitination can be removed by UCHL1.

To further explore the ubiquitination site of TAZ, we predicted the site on the PLMD website and found that only one lysine site of TAZ could be ubiquitinated (K46). Residue K46 on TAZ was conserved across most mammals (Figure 4E). To elucidate the role of the K46 residue on TAZ, we generated a point mutation in TAZ by replacing lysine with alanine (K46A). Compared with wild-type TAZ, the TAZ mutant (K46A) can resist the decreased protein expression caused by knockdown of UCHL1 and increase the stability of TAZ (Figure 4F and G). Co-IP assays also showed a weakened interaction between Ubi and the TAZ mutant (K46A), confirming that K46 on TAZ was essential for UCHL1 binding with TAZ and further regulation of its degradation (Figure 4H). Together, these data indicate that UCHL1 is specifically involved in the K48-linked deubiquitination of TAZ and that K46 is the ubiquitination site of TAZ.

### TAZ regulates NFATC1 through a nontranscriptional coactivator function

**Figure 5:**
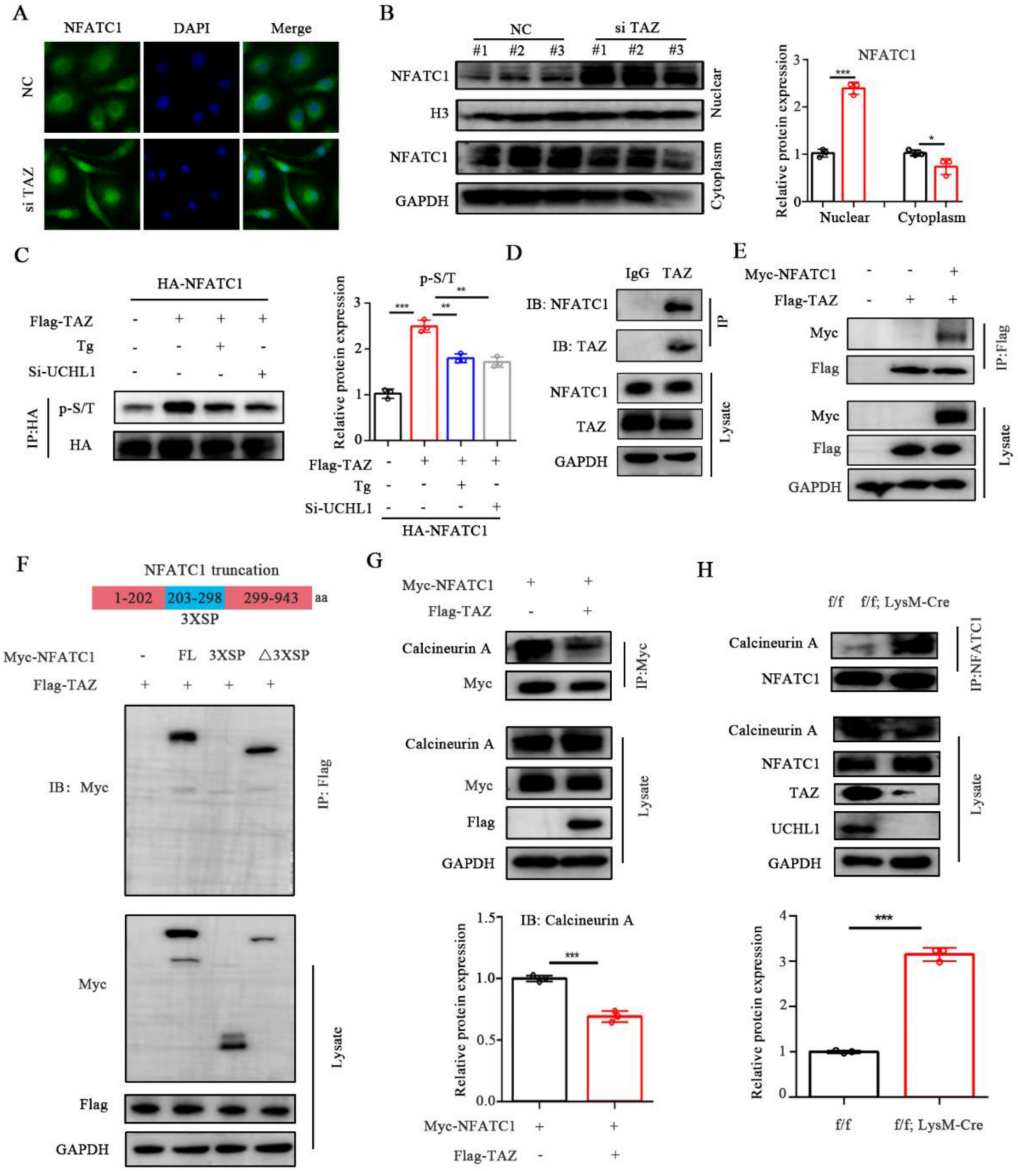
TAZ competes with CNA for binding to NFATC1. A) Microscope images of NFATC1 immunofluorescence of BMMs transfected with NC or si-TAZ for 48 hours. Scale bars, 10 μm. B) The protein levels of NFATC1 in nucleus and cytoplasm of BMMs with TAZ knockdown. C) Phosphorylation level of NFATC1 in TAZ overexpressed HEK-293T cells after 1 mM Tg treatment or UCHL1 knockout. D) Endogenous protein interaction between TAZ and NFATC1 in BMMs was examined by immunoprecipitate with anti-rabbit IgG or anti-TAZ antibody, and analyzed by WB with antibodies to detect NFATC1 and TAZ. E) Exogenous protein interaction between TAZ and NFACT1 was demonstrated in HEK-293T. F) Full-length and various truncated fragments of NFATC1. FL, 3XSP, △3XSP and TAZ plasmids were co-transfected in HEK-293T cells. The interactions were detected through CO-IP analyses. G) Changes in binding capacity of NFATC1 to Calcineurin (CNA) after overexpression of TAZ by using Co-IP experiment. H) Changes in binding ability of NFATC1 to Calcineurin (CNA) in f/f; LysM-Cre BMMs by using Co-IP experiment. Data are presented as mean ± SD. *P < 0.05, **P < 0.01, ***P < 0.001.

NFATC1 is a key cytokine in the process of osteoclast differentiation and is dephosphorylated into the nucleus by calcineurin, thereby promoting the transcription and expression of osteoclast-related genes. To investigate the effect of TAZ on NFATC1, we transfected TAZ-siRNA or si-NC into BMM cells and observed that knockdown of TAZ resulted in increased nuclear entry of NFATC1 (Figure 5A). We then evaluated the expression of NFATC1 in the nucleus and cytoplasm of TAZ knockdown BMMs. As shown in Figure 5B, the expression of NFATC1 was significantly increased in the nucleus of TAZ knockdown BMMs, suggesting that knockdown of TAZ could promote nuclear translocation of NFATC1, which was consistent with the immunofluorescence results.

Dephosphorylation of NFATC1 affects its nuclear transport. To further explore whether TAZ regulates the phosphorylation level of NFATC1, HEK-293T cells were transfected with HA-NFACT1 and/or Flag-TAZ overexpression plasmids. Coimmunoprecipitation results illustrated that phosphorylation of NFATC1 was increased after the addition of TAZ but inhibited after the addition of thapsigargin (Tg, which activates calcineurin and promotes dephosphorylation of NFATC1 into the nucleus). Moreover, the TAZ-induced enhancement of NFATC1 phosphorylation was also inhibited by UCHL1 knockdown (Figure 5C). These data suggest that TAZ controls NFATC1 nuclear translocation through a calcineurin-dependent phosphorylation modification process.

TAZ regulates phosphorylation and nuclear transport of NFATC1, which prompted us to hypothesize that there is an interaction between TAZ and NFATC1. Furthermore, co-IP confirmed that NFATC1 was significantly precipitated by the anti-TAZ antibody in BMMs (Fig. 5D). Exogenous overexpression assays showed that Myc-tagged NFATC1 was also highly coprecipitated with flag-tagged TAZ (Fig. 5E). These results suggest that TAZ interacts with NFATC1.

Phosphorylation sites of NFATC1 are located in multiple serine-rich motifs in the regulatory domain, including SER-Pro-X-X repeat motifs SP1, SP2, and SP3 (3 X SP) 34. To characterize the key region where TAZ binds to NFATC1, we constructed NFATC1-3XSP and NFATC1-△3XSP plasmids according to the protein domain information of NFATC1 in the UniProt website (the 3XSP region is the key region of NFATC1 phosphorylation). Subsequently, HEK-293T cells were cotransfected with Flag-TAZ and full-length Myc-NFATC1 or the two truncated plasmids. Immunoprecipitation results illustrated that TAZ binds to NFATC1 without the 3XSP region, suggesting that TAZ does not regulate phosphorylation and nuclear transport by directly binding to the phosphorylation region of NFATC1 (Figure 5F). We suspected the potential participation of TAZ in NFATC1 dephosphorylation. By overexpressing TAZ and NFATC1 in HEK-293T cells, we found that TAZ reduced the binding ability of NFATC1 to calcineurin A (CNA) (Figure 5G). In cKO BMMs, the binding capacity of NFATC1 to calcineurin A was enhanced (Figure 5H). These results indicated that TAZ regulated by UCHL1 does not function as a transcriptional coactivator but competes with CNA for binding to NFATC1, resulting in upregulation of NFATC1 phosphorylation and reduced nuclear entry.

### UCHL1 protects mice from established bone loss in various pathological models

**Figure 6:**
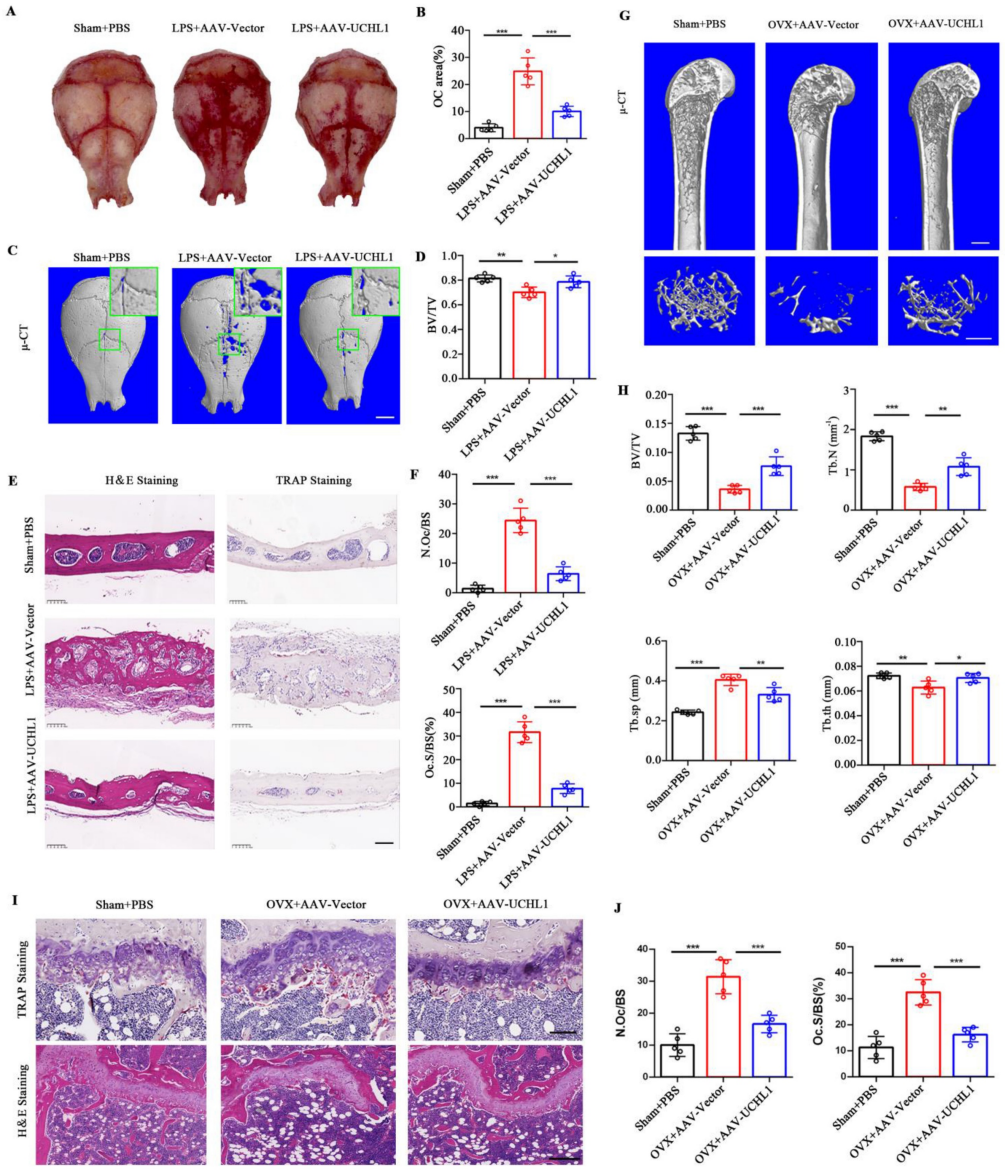
UCHL1 protects mice from established acute and chronic bone loss. A) Gross view of TRAP-stainded calvaria. B) Quantification of relarive TRAP-positive area in calvaria. C) μ-CT and 3D images of calvaria Sham or LPS operated mice injected with AAV-Vector or AAV-UCHL1. Scale bar, 1 mm. D) Quantification of bone BV/TV of indicated groups (n = 5). E, F) H&E, TRAP staining and corresponding quantitative analysis of calvaria from the three groups. Scale bars, 100 μm. G) μ-CT and 3D images of Sham or OVX operated mice injected with AAV-Vector or AAV-UCHL1. Scale bar, 1 mm. H) Quantification of BV/TV, Tb. N, Tb. Sp, and Tb. Th from the three groups (n = 5). I) TRAP and H&E staining of femora from the three groups. Scale bars, 100 μm. J) Quantification of osteoclast number/bone surface (N. Oc/BS) and the percentage of osteoclast surface per bone surface (Oc. S/BS (%)) (n=5). Data are presented as mean ± SD. *P < 0.05, **P < 0.01, ***P < 0.001.

To investigate the potential clinical significance of UCHL1, we used adeno-associated virus (AAV) gene expression to locally overexpress UCHL1 in mice with acute and chronic bone loss models. GFP fluorescence was used to detect successful AAV infection *in vivo* (Supplementary Figure 5). Lipopolysaccharide (LPS)-induced acute osteolysiswas used as a model for acute bone loss. Overall, the LPS-induced osteoclast area was significantly increased and the Trap-positive area was decreased in the AAV-UCHL1 treatment group compared with the local injection of PBS group (Figure 6A and B). Micro-CT scans showed that bone surface porosity and bone solubility decreased and BV/TV increased after UCHL1-AAV treatment (Figure 6C and D). H&E staining showed that the LPS+Vector group had rougher surfaces and more osteolysis than the sham group, while local administration of AAV-UCHL1 reduced inflammatory cell infiltration and bone erosion (Figure 6E, left panel). TRAP staining further confirmed that local overexpression of UCHL1 reduced osteoclast activity after inflammatory stimulation (Figure 6E and F).

An ovariectomized (OVX) animal model of osteoporosis was used for the chronic bone loss model. As assessed by u-CT, application of AAV-UCHL1 showed a restoration of OVX-induced osteoporosis by increasing BV/TV, Tb. N and Tb. Th and decreasing Tb. Sp compared with the AAV-Vector-treated group (Figure 6G and H). Furthermore, the protective effect of AAV-UCHL1 administration on OVX-induced chronic bone loss was confirmed by TRAP and H&E staining (Figure 6I and J). In conclusion, overexpressing UCHL1 *in vivo* to target pathologic bone loss is possible.

## Discussion

The abnormal differentiation and function of osteoclasts are closely related to the occurrence of osteoporosis, and an in-depth understanding of the mechanism of osteoclastogenesis could provide multiple options for the treatment of physiological and pathological bone loss 35. UCHL1 is a DUB that has been shown to be involved in regulating cancer, neurodegenerative diseases, and cardiac hypertrophy by removing the ubiquitination of multiple proteins 10, 36, 37. However, the role of UCHL1 in osteoclasts has not been reported. Here, we discovered that UCHL1 regulates osteoclast formation and resorptive activity by maintaining TAZ stability.

In this study, comprehensive transcriptome and proteomic analysis showed that UCHL1 was the most differentially expressed deubiquitination enzyme during osteoclast differentiation. We first demonstrated that UCHL1 expression increased over time during osteoclast differentiation *in vitro*, and knockdown of UCHL1 in osteoclast precursors promoted osteoclast formation and bone resorption. We then constructed LysM-Cre-UCHL1f/f mice and found that osteoclast-specific deletion of UCHL1 resulted in severe osteoporosis under pathological conditions, suggesting that UCHL1 exerted an inhibitory effect on osteoclast hyperactivation. Shim et al. used gracile axonal dystrophy (gad) mice that mimic global UCHL1 knockout, leading to reduced bone mineralization and bone mass. This is consistent with the phenotype of osteoporosis in mice after osteoclast-specific knockout of UCHL1.

In search of the substrate protein responsible for UCHL1 regulation in osteoclastogenesis, we identified TAZ through a literature review and immunoprecipitation. Here, we provided convincing evidence that UCHL1 affected the protein expression of TAZ, while the mRNA level of TAZ did not change, indicating that UCHL1 is involved in the posttranslational modification of TAZ rather than regulation at the transcriptional level. Using Co-IP, we identified the WW domain of TAZ (amino acids 101-200) that aided the interaction between UCHL1 and TAZ.

TAZ is a key downstream effector of the Hippo signalling pathway and is a transcriptional coactivator that interacts with multiple transcription factors to regulate the growth of muscles, embryos, lungs, limbs, and hearts 38-41. Current studies on bone metabolism have mainly proven that TAZ promotes the differentiation and function of osteoblasts 42. Overexpression of TAZ in osteoblasts increased bone mass, while osteoblast-specific knockout of TAZ led to an osteoporotic phenotype in mice 43, 44. TAZ global knockout (gKO) mice have a partial probability of death, and only mild skeletal deformities and abnormalities are found in surviving mice 45, 46. Qian et al. constructed osteoclast conditional TAZ knockout mice and demonstrated an osteoporotic phenotype in TAZ cKO mice, which was consistent with that of UCHL1 cKO mice 24. To further clarify the relationship between TAZ and UCHL1 in osteoclast differentiation, we transfected TAZ-overexpressing adenovirus into UCHL1 cKO BMMs and found that TAZ could inhibit the promotion of osteoclast differentiation by knockout of UCHL1, suggesting that TAZ is a downstream protein of UCHL1 regulating osteoclast differentiation. However, the mechanisms underlying the correlation between UCHL1 and TAZ remain to be explored.

Ubiquitination modifications degrade substrate proteins; K48, K11 and the linear ubiquitin chain (M1) have this function, while the main function of K63 is to localize proteins to lysosomes, thereby regulating protein signal transduction and other functions^47^. Our data identified the K48 polyubiquitin chain involved in UCHL1 deubiquitination of the TAZ protein, which is consistent with the results that UCHL1 inhibits TAZ degradation.

**Figure 7:**
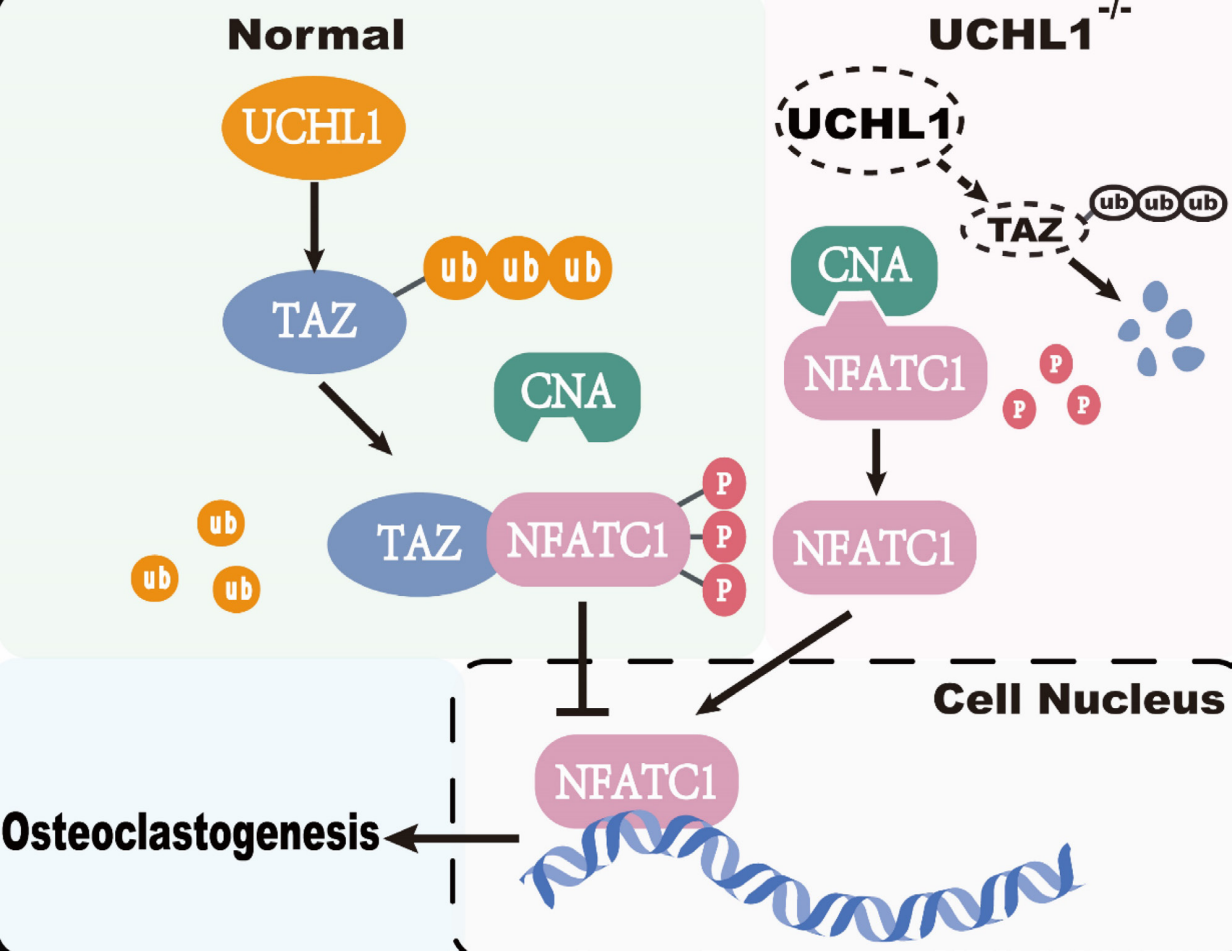
Working model of UCHL1 as a negative regulator in osteoclast.

Current studies have shown that TAZ functions not only as a transcriptional coactivator but also as a nontranscriptional coactivator by binding to proteins. YAP/TAZ binds directly to TANK binding kinase 1 (TBK1) independent of transcriptional level regulation and eliminates virus-induced TBK1 by preventing TBK1 lys63-linked ubiquitination and linker/substrate binding activation 48. TAZ acts as a corepressor of PPARγ, regulates PPARγ dephosphorylation and inhibits adipogenesis 21. In our study, TAZ competes with CNA for binding to NFATC1, which inhibits NFATC1 dephosphorylation and nuclear transport. Calcineurin consists of a calcineurin A (CNA) catalytic subunit and a Ca2+-binding calcineurin B (CNB) subunit 49. CNA includes a calmodulin-binding (CaM-binding) domain and an autoinhibitory domain (AID). Once CNB and CaM bind to Ca2+, the structure of AID changes, thus releasing its inhibitory effect on CNA catalytic activity. NFATC1 is the substrate of CNA, and activated CNA directly dephosphorylates NFATC1 in the cytoplasm and induces its translocation to the nucleus 50, 51. The critical role of NFATC1 activity in osteoclastogenesis is well documented 52. Our co-IP results showed that TAZ decreased the binding capacity of NFATC1 to CNA, and subsequently, toxic carotene (calcium agonist) rescued this process, resulting in reduced phosphorylation of NFATC1. Unfortunately, as a downstream target of UCHL1, TAZ competes with CNA for binding to NFATC1, which inhibits NFATC1 dephosphorylation and nuclear transport to impede osteoclastogenesis (Figure 7).

Notably, our results from both loss-of-function and gain-of-function experiments demonstrated that UCHL1 is an important regulator of osteoclastogenesis. In conclusion, activating UCHL1 may serve as a novel therapeutic approach targeting bone loss in various bone pathological states.

## Supplementary Material

Supplementary figures and tables.
