# Supplementary material for: The deubiquitinase UCHL1 negatively controls osteoclastogenesis by regulating TAZ/NFATC1 signalling
Source: Int J Biol Sci. 2023 Apr 24;19(8):2319–32. doi: 10.7150/ijbs.82152 (PMC10197889; doi:10.7150/ijbs.82152)
Supplement: Supplementary file 1 — Supplementary figures and tables. [file ijbsv19p2319s1.pdf]

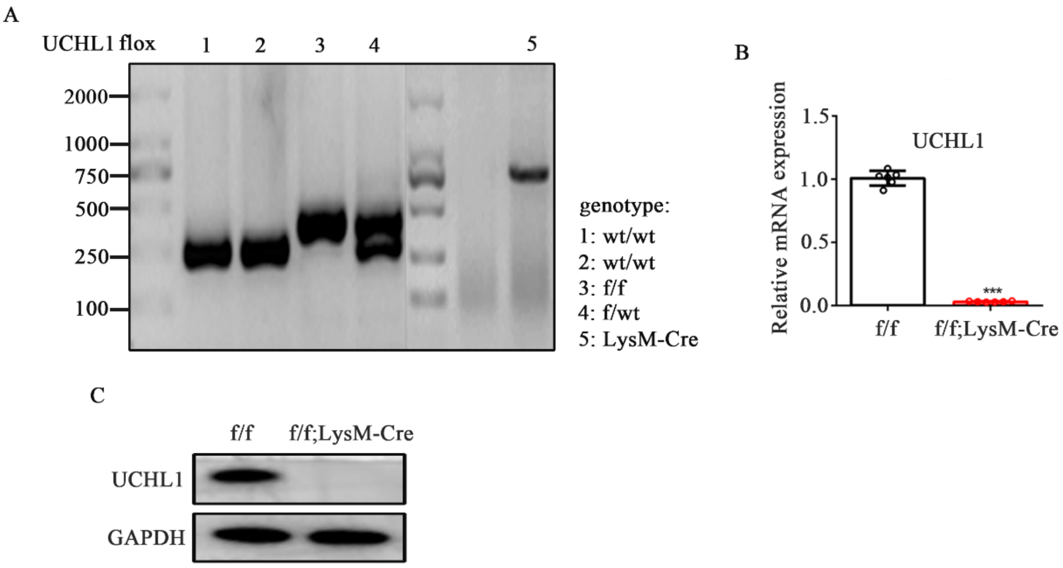

Supplementary Figure 1. UCHL1 conditional knock out (cKO) mice. A) Genotyping of UCHL1 wt/wt, UCHL1 flox/flox, UCHL1 flox/wt, LysM-Cre mice determined by PCR. B) Efficiency of UCHL1 knockout in UCHL1 flox/flox; LysM-cre BMMs detected by RT-qPCR. C) Efficiency of UCHL1 knockout in UCHL1 flox/flox; LysM-cre BMMs detected by WB. Data are presented as mean ± SD. *P < 0.05, **P < 0.01, ***P < 0.001.

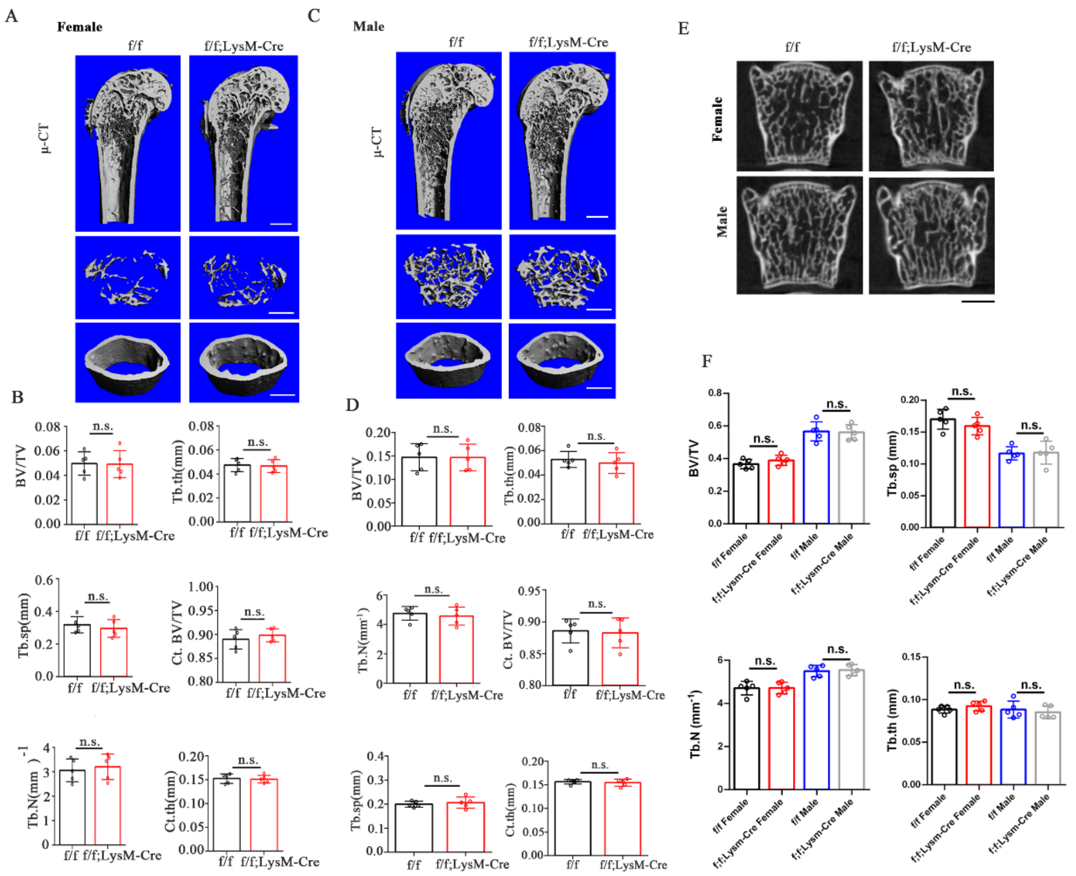

Supplementary Figure 2. UCHL1 cKO has no influence on bone mass under Physiological condition. A, C) Micro-CT images of the proximal femur from female and male flox/flox and UCHL1 cKO mice. Scale bars, 1 mm. B, D) Quantification of bone volume per tissue volume (BV/TV), trabecular separation (Tb. Sp), trabecular number (Tb. N), trabecular thickness (Tb. Th), cortical region BV/TV (Ct. BV/TV) and cortical thickness (Ct. Th, mm) (n = 5). E) Coronal images of the second lumbar spine. Scale bars, 1 mm. F) Quantification of trabecular bone parameters of lumbar spine (n = 5). Data are presented as mean ± SD. *P < 0.05, **P < 0.01, ***P < 0.001.

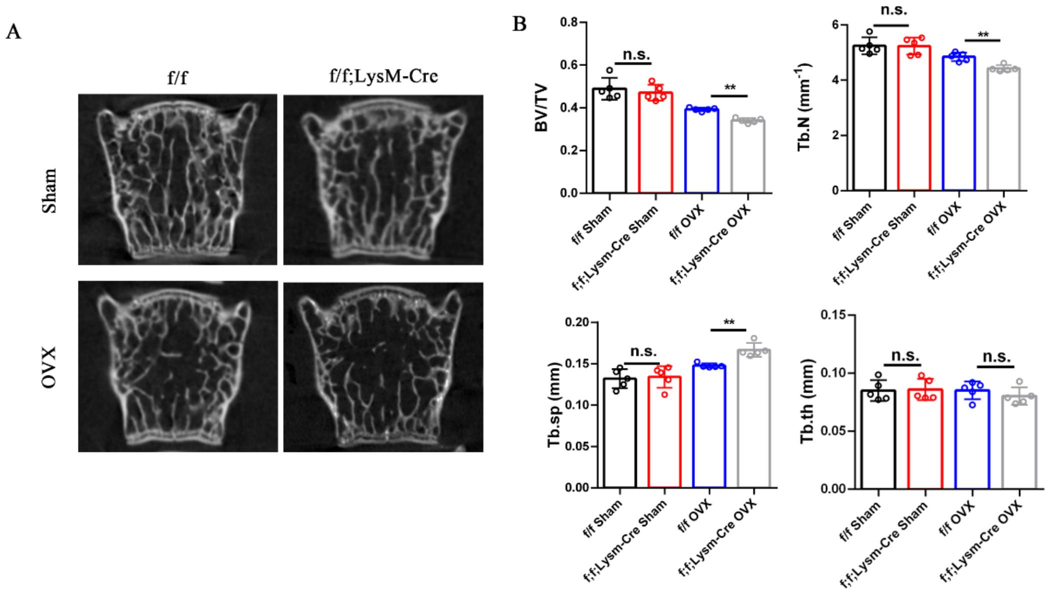

Supplementary Figure 3. UCHL1 deletion results in severe osteoporosis in axial skeleton of the OVX model. A) Coronal images of the second lumbar spine. Scale bars, 1 mm. B) Quantification of trabecular bone parameters of lumbar spine (n = 5). Data are presented as mean ± SD. *P < 0.05, **P < 0.01, ***P < 0.001.

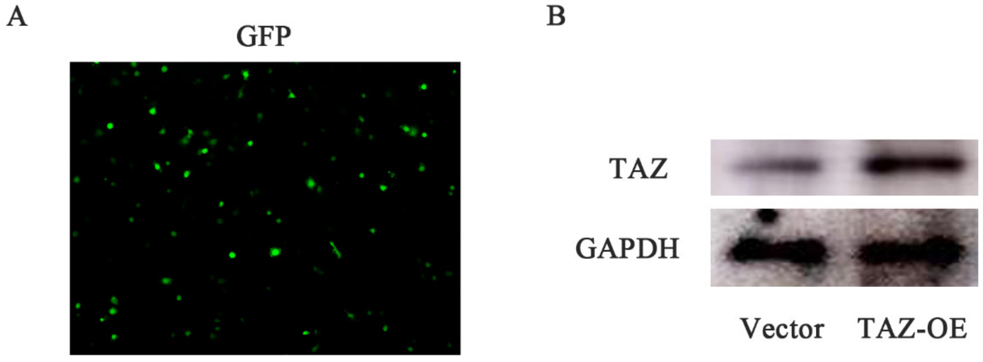

Supplementary Figure 4. Analysis of the expression of UCHL1 overexpression adenovirus. A) BMMs were transfected with GFP-tagged UCHL1 overexpressing adenovirus, and GFP was observed 48 hours post-transfection. B) Expression of UCHL1 active form (Gα13CA) in BMMs was confirmed by western blot.

A

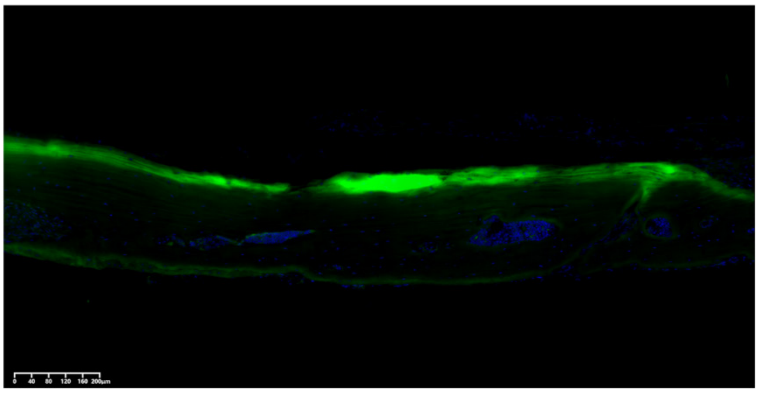

B

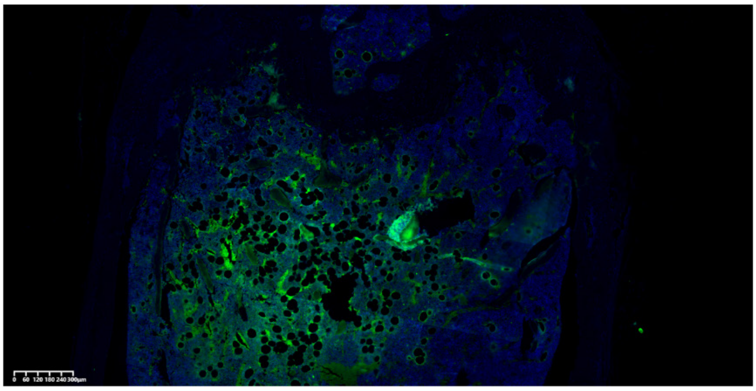

Supplementary Figure 5. Analysis of the effective infection of AAV in the cranium and femur. A) Image of GFP-UCHL1-AAV in cranium. Scale bars, 200 μm. B) Image of GFP-UCHL1-AAV in femur. Scale bars, 300 μm.

Supplementary Table 1. Primers and sequences used in this study.

Primers for qPCR		
GAPDH	F	GGAGAGTGTTTCCTCGTCCC
	R	ATGAAGGGGTCGTTGATGGC
NFATC1	F	CCCGTCACATTCTGGTCCAT
	R	CAAGTAACCGTGTAGCTGCACAA
CTSK	F	GCTCACAGTAGCCACGCTT
	R	AACGCCGAGAGATTTCATCCA
C-fos	F	GGGAATGGTGAAGACCGTGT
	R	CCGTTCCCTTCGGATTCTCC
Dcstamp	F	TTTCCACGAAGCCCTAGCTG
	R	GCGTTCCTACCTTCACGGAG

Acp5	F	AAGAGATCGCCAGAACCGTG
	R	TTCCAGCCAGCACATACCAG
Atp6v0d2	F	CAGAGCTGTACTTCAATGTGGAC
	R	AGGTCTCACACTGCACTAGGT
UCHL1	F	GATGCTGAACAAAGTGTTGGC
	R	GGAGTTTCCGATGGTCTGCTT
WWTR1	F	CATGGCGGAAAAAGATCCTCC
	R	GTCGGTCACGTCATAGGACTG
